# Liver stiffness-spleen size-to-platelet ratio risk score detects esophageal varices in chronic liver disease

**DOI:** 10.1186/s40064-016-2708-1

**Published:** 2016-07-07

**Authors:** Soichiro Shibata, Satoru Joshita, Takeji Umemura, Tomoo Yamazaki, Naoyuki Fujimori, Yuki Ichikawa, Michiharu Komatsu, Akihiro Matsumoto, Eiji Tanaka

**Affiliations:** Division of Hepatology and Gastroenterology, Department of Medicine, Shinshu University School of Medicine, 3-1-1 Asahi, Matsumoto, 390-8621 Japan

**Keywords:** LSPS, Transient elastography, CLD, Esophageal varices

## Abstract

**Background:**

Noninvasive markers are needed to identify esophageal varices (EV) in patients with chronic liver disease (CLD). Recently, liver stiffness (LS)-spleen size-to-platelet ratio risk score (LSPS) has been shown to predict EV in patients with chronic hepatitis C. The aim of this study was to validate the clinical value of LSPS for EV detection and identification of high risk EV in Japanese patients with CLD.

**Methods:**

A total of 230 patients with CLD who had undergone endoscopy, LS measurement, and ultrasonography between 2013 and 2015 were enrolled. The relationships between clinical data and LSPS were compared with those for other noninvasive markers (aspartate aminotransferase-to-platelet ratio, FIB-4 index, and platelet-to-spleen ratio), along with platelet count, spleen size, and LS. Diagnostic and prognostic abilities were assessed by the area under the receiver operating characteristic curve (AUC) and multivariate logistic regression.

**Results:**

LSPS correlated significantly with EV grade (*P* < 0.001) and was superior to the other noninvasive indices for determination of EV and high risk EV. Furthermore, LSPS was independently associated with the presence of EV (*P* < 0.001) and elevated EV risk (*P* = 0.013) by multivariate logistic regression analysis. The optimal cutoff values of LSPS for EV and high risk EV were 1.1 and 2.2, respectively, at which AUC, negative predictive value, and accuracy were 0.821 [95 % confidence interval (CI) 0.743–0.899], 91.9, and 84.3 % and 0.859 (95 % CI 0.736–0.981), 95.5, and 76.9 %, respectively.

**Conclusions:**

LSPS represents a useful, noninvasive, accurate method to detect EV and a high EV risk in Japanese patients with CLD.

**Electronic supplementary material:**

The online version of this article (doi:10.1186/s40064-016-2708-1) contains supplementary material, which is available to authorized users.

## Background

Esophageal varices (EV) are the most relevant porto-systemic collaterals resulting from clinically significant portal hypertension, for which the presence of EV is an independent predictor of mortality (D’Amico et al. 2006). As acute variceal bleeding is a major complication of cirrhosis, patients with newly diagnosed cirrhosis in chronic liver disease (CLD) are advised to undergo endoscopic screening for EV (de Franchis and Baveno 2010). However, endoscopy is an invasive and unpleasant procedure that carries rare, but serious, complications. Thus, simple, noninvasive, accurate tests are needed to predict EV in CLD. The liver stiffness (LS)-spleen size-to-platelet ratio score (LSPS), which is a combination of 3 simple examination methods (LS, spleen size, and platelet count) has been found to predict EV and high risk EV in patients with compensated cirrhosis (Berzigotti et al. 2013; Kim et al. 2010). We also recently reported that LSPS could identify EV in Japanese patients with chronic hepatitis C virus (HCV) infection (Shibata et al. 2015). Since this method has not yet been validated for other etiologies of CLD in Japan, the present investigation evaluated the ability of LSPS to predict the presence of EV and high risk EV in Japanese patients with CLD.

## Methods

### Subjects

**Fig. 1:**
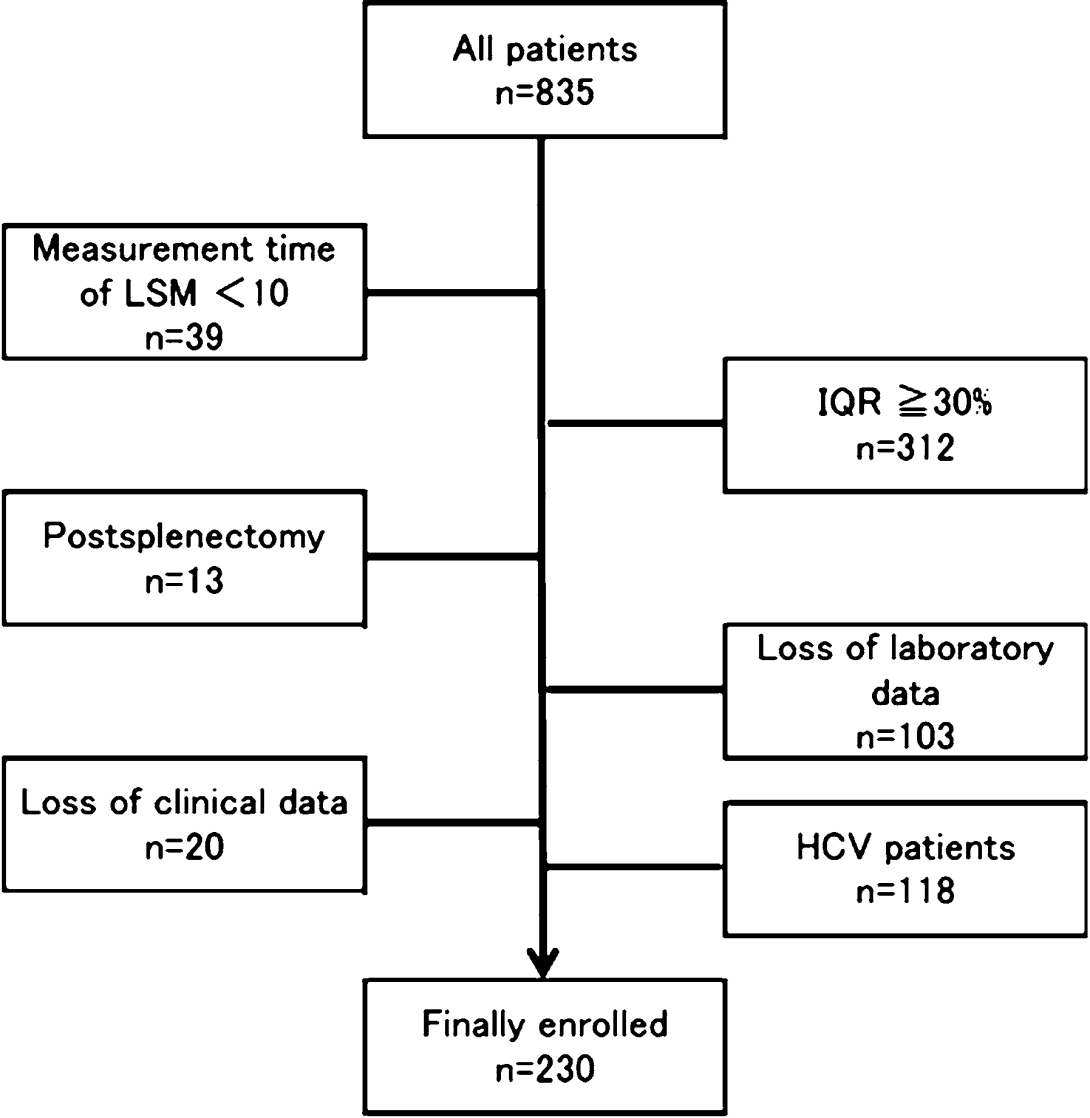
Flow-chart of all patients enrolled in this study. A total of 835 consecutive patients with CLD who were evaluated by endoscopy, LS measurement, and ultrasonography within an interval of 6 months and without a history of variceal bleeding and ascites were included. Following the exclusion criteria, 230 patients with CLD were selected and analyzed in this study

A total of 835 consecutive patients with CLD who were seen at Shinshu University Hospital (Matsumoto, Japan) between April 2013 and December 2015 and evaluated by endoscopy, LS measurement, and ultrasonography within an interval of 6 months and without a history of variceal bleeding or ascites were recruited. Exclusion criteria were as follows: (1) fewer than 10 LSM measurements, (2) IQR of greater than 30 %, (3) receiving splenectomy, (4) lack of laboratory data, (5) lack of clinical data, and (6) HCV infection [the data on which have already been published (Shibata et al. 2015)], as presented in Fig. 1. Ultimately, a total of 230 patients with CLD were enrolled in this retrospective, cross-sectional study. The diagnosis of CLD was based on the following disease criteria: hepatitis B surface antigen and hepatitis B virus (HBV) DNA in patients positive for hepatitis B surface antigen were evaluated to identify persistent HBV infection (Umemura et al. 2008). Autoimmune hepatitis (Umemura et al. 2007) and primary biliary cirrhosis (Umemura et al. 2012) were diagnosed by histological examination and serological testing, as previously reported. Alcoholic liver disease and non-alcoholic fatty liver disease (NAFLD) were determined using conventional methods. Non-alcoholic steatohepatitis (NASH) was diagnosed by histological examination (Kleiner et al. 2005) (Additional file 1: Figure S1).

### Research ethics considerations

This study was conducted in accordance with the principles of the 1975 Declaration of Helsinki. The protocol of this investigation was reviewed and approved by the Shinsu University ethics committee (number 3021). Informed consent was obtained from all participants.

### Laboratory testing

All laboratory data were obtained on the same day as transient elastography (TE) scanning. Alanine aminotransferase, aspartate aminotransferase, and other relevant biochemical tests were performed using standard methods. Four surrogate blood indices of liver fibrosis and EV prediction were also assessed at enrollment according to published formulae: aspartate aminotransferase-to-platelet ratio index (APRI) (Wai et al. 2003) and FIB-4 index (Sterling et al. 2006) were calculated as: (AST/upper limit of normal; 40 IU/l) × [100/platelet count (10^9^/l)] and [age (years) × AST (IU/l)]/[platelet count (10^9^/l) × ALT (IU/l)^1/2^], respectively. The platelet-to-spleen ratio (Giannini et al. 2003) and LSPS (Berzigotti et al. 2013; Kim et al. 2010) formulae were as follows: platelet count/spleen diameter and LS value × spleen diameter/platelet count, respectively.

### Ultrasonography (US) and TE

US and TE were performed on the same day following overnight fasting. Spleen size was measured on US images before TE as spleen bipolar diameter (i.e., crossing the spleen hilium) with a convex probe. The experienced US operators were unaware of the patients’ clinical details.

TE was determined with a FibroScan device (FibroScan502, Echosens, Paris, France) using a 50 Hz standard M probe, as previously described (Sandrin et al. 2003). All patients were assessed using a 3.5 MHz standard M probe (EUB-8500, Hitachi Medical Co., Ltd., Japan). LS was determined as the median value of 10 measurements and expressed in kilopascals (kPa).

### Endoscopy for EV

Two experienced endoscopists who were blinded to the LS results performed all endoscopic examinations. EV staging was classified as none (no veins above the esophageal mucosal surface; F0), small (minimally elevated veins above the esophageal mucosal surface; F1), medium (large tortuous veins occupying <1/3 of the lumen; F2), or large (large coil-shaped veins occupying ≥1/3 of lumen; F3) according to standard criteria published by the Japan Society for Portal Hypertension (Beppu et al. 1981). The high risk EV group included all patients with F2/F3 EV or those having F1 EV with red color signs.

### Statistical analysis

Categorical variables were compared using the Chi squared or Fisher’s exact tests, as appropriate. Continuous variables were assessed by the Mann–Whitney *U* or Kruskal–Wallis tests, and abnormally distributed variables were determined by the Shapiro–Wilk test. Diagnostic accuracy was calculated using receiver operating characteristic (ROC) curve analysis in terms of sensitivity, specificity, positive predictive value (PPV), negative predictive value (NPV), and area under the ROC curve (AUC). Cutoff values were identified by the Youden index, and the nearest clinically applicable value to the cutoff was considered as the optimal cutoff value for clinical convenience. Multivariate forward stepwise logistic regression analysis was employed to identify independent factors predictive of the absence or presence of EV and high risk EV.　Comparisons of paired AUCs and 95 % confidence intervals (CIs) were carried out using the nonparametric Delong test. A *P* < 0.05 was considered to be statistically significant. Statistical analyses were performed using IBM SPSS Statistics ver. 21.0 (IBM, Chicago, IL) and StatFlex ver. 6.0 (Artech Co., Ltd. Osaka, Japan) software.

## Results

### Baseline clinical characteristics of patients

The baseline characteristics of the 230 patients are summarized in Table 1. Median age was 63 years and male incidence was 49 %. The main etiology of CLD was NAFLD/NASH (25 %), followed next by HBV (20 %), primary biliary cirrhosis (18 %), alcoholic liver disease (10 %), autoimmune hepatitis (7 %), and others (20 %), such as cryptogenic cirrhosis, Budd-Chiari syndrome, hemochromatosis, primary sclerosing cholangitis, and drug-induced liver injury. One hundred and ninety-one patients had no EV, 31 had F1 EV (2 with red color signs), 7 had F2 (4 with red color signs) and 1 had F3 and red color signs. Overall, 29 patients belonged to the low risk EV group and 10 belonged to the high risk EV group. We conducted an additional sub-analysis in terms of etiology, which included alcoholic LD (n = 23), and found that these patients displayed a significantly higher LS than did others (*P* < 0.001 as compared with each etiology). However, since the number of alcoholic LD subjects was too small to make a definitive conclusion, we conducted the study to include all etiologies apart from HCV.

**Table 1:** Baseline characteristics of 230 CLD patients

Characteristic	Total (n = 230)
Age (years)	63 (10–90)
Male gender	113 (49)
Body mass index (kg/m^2^)	22.7 (11.9–41.1)
Etiology	
NAFLD/NASH	57 (25)
HBV	47 (20)
PBC	42 (18)
ALD	23 (10)
AIH	15 (7)
Other	46 (20)
Platelet count (×10^9^/l)	182 (14–713)
AST (IU/l)	30 (6–1261)
ALT (IU/l)	28 (6–1246)
Albumin (g/dl)	4.1 (1.4–4.9)
T.Bil (mg/dl)	0.8 (0.2–24.2)
PT%	92 (24–128)
LS (kPa)	5.9 (1.5–75.0)
Spleen size (cm)	8.9 (3.9–24.0)
LSPS	0.3 (0.0–20.0)
APRI	0.5 (0.1–33.7)
FIB-4	1.9 (0.1–25.5)
Platelet-to-spleen ratio	2091 (166–7809)

Values are expressed as median (range, minimum–maximum) or n (%)

### Diagnostic performance of noninvasive methods for EV

**Fig. 2:**
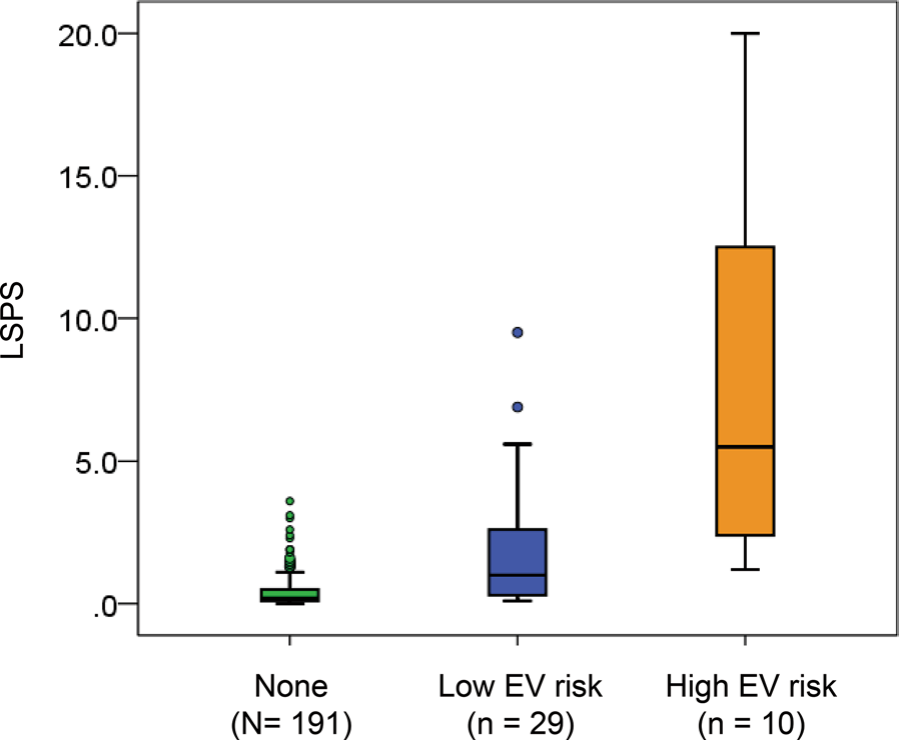
LSPS detection of the presence and high risk EV. *Boxes* represent the IQR of the data. The *lines across the boxes* indicate median values. The *hash marks above and below the boxes* indicate the 90th and 10th percentiles for each group, respectively. *Open circles* indicate outliers

The clinical characteristics of patients with and without EV are shown in Table 2. Total bilirubin was significantly higher in patients with EV than in those without, while platelet count, albumin, and PT% were significantly lower. All noninvasive markers (LSPS, APRI, FIB-4, and platelet-to-spleen ratio) were significantly associated with EV (all *P* < 0.001). Multivariate analysis demonstrated that LSPS [odds ratio (OR) 2.441, 95 % CI 1.747–3.413; *P* < 0.001] was independently associated with the presence of EV. As shown in Fig. 2, median LSPS values were 0.2 (IQR 0.1–0.5), 1.0 (IQR 0.3–2.9), and 5.5 (IQR 2.4–12.5) in the no EV, low risk EV, and high risk EV groups, respectively. LSPS values were significantly correlated with EV severity (*P* < 0.001, Kruskal–Wallis test).

**Table 2:** Comparison between patients with and without EV

Characteristic	EV (+) (n = 39)	EV (−) (n = 191)	*P* value
Age (years)	61 (13–90)	63 (10–87)	0.553
Male gender	25 (64)	88 (46)	0.040
Body mass index (kg/m^2^)	24.5 (17.3–41.1)	22.7 (11.9–41.0)	0.037
Etiology			
NAFLD/NASH	7 (18)	50 (26)	0.378
HBV	10 (26)	37 (19)	0.377
PBC	5 (13)	37 (19)	0.461
ALD	10 (26)	13 (7)	0.001
AIH	0 (0)	15 (8)	0.146
Other	7 (18)	39 (20)	0.895
Platelet count (×10^9^/l)	99 (34–683)	197 (14–713)	0.013
AST (IU/l)	33 (15–146)	29 (6–1261)	0.194
ALT (IU/l)	25 (8–123)	28 (6–1246)	0.196
Albumin (g/dl)	3.7 (2.7–4.6)	4.2 (1.4–4.9)	0.005
T.Bil (mg/dl)	1.2 (0.4–7.5)	0.7 (0.2–24.2)	<0.001
PT%	74 (27–95)	95 (24–128)	<0.001
LS (kPa)	10.0 (3.5–75.0)	5.3 (1.5–75.0)	<0.001
Spleen size (cm)	11.4 (6.1–24.0)	8.6 (3.9–14.8)	<0.001
LSPS	1.7 (0.1–20.0)	0.2 (0.0–6.4)	<0.001
APRI	1.4 (0.1–4.8)	0.5 (0.1–33.7)	<0.001
FIB-4	5.7 (0.2–12.0)	1.8 (0.1–25.5)	<0.001
Platelet-to-spleen ratio	770 (220–5991)	2201 (166–7809)	<0.001

Values are expressed as median (range, minimum–maximum) or n (%)

We next performed ROC curve analysis to determine the predictive accuracy of the noninvasive parameters for EV in CLD. The values for AUC, optimal cutoff value, sensitivity, specificity, PPV, NPV, and accuracy for the presence of EV are listed in Table 3. AUCs were 0.821 for LSPS, 0.807 for platelet-to-spleen ratio, 0.800 for platelet count, 0.779 for FIB-4 index, 0.775 for spleen size, 0.765 for LS, and 0.749 for APRI. Although LSPS had the highest discrimination for EV, there were no significant differences between the AUC of LSPS and those for platelet-to-spleen ratio, platelet count, FIB-4, spleen size, LS, or APRI. An LSPS cutoff value of 1.1 yielded a sensitivity of 61.5 %, specificity of 89.0 %, PPV of 53.3 %, NPV of 91.9 %, and accuracy of 84.3 % (Table 3).

**Table 3:** Performance of noninvasive parameters for identifying EV

Characteristic	Cutoff	AUC (95 % CI)	Sensitivity (%)	Specificity (%)	PPV (%)	NPV (%)	Positive Likelihood ratio	Negative Likelihood ratio	Accuracy (%)
LSPS	1.1	0.821 (0.743–0.899)	61.5	89.0	53.3	91.9	5.60	0.43	84.3
Platelet count (×10^9^/l)	14.3	0.800 (0.708–0.892)	79.5	79.6	44.3	95.0	3.89	0.26	79.6
Platelet-to-spleen ratio	1330	0.807 (0.712–0.901)	76.9	84.3	50.0	94.7	4.90	0.27	83.0
Spleen size (cm)	9.8	0.775 (0.697–0.852)	74.4	79.1	42.0	93.8	3.55	0.32	78.3
LS (kPa)	6.1	0.765 (0.688–0.843)	84.6	61.3	30.8	95.1	2.18	0.25	65.2
APRI	1.2	0.749 (0.655–0.844)	59.0	84.8	44.2	91.0	3.88	0.48	80.4
FIB-4 index	4.1	0.779 (0.690–0.868)	61.5	89.5	54.5	91.9	5.88	0.43	84.8

### Prediction of high risk EV in 39 patients with EV

Among the 39 patients complicated with EV, total bilirubin was significantly higher in patients with high risk EV than in those without, while platelet count and PT% were significantly lower. Among the noninvasive markers, patients with high risk EV had significantly higher LSPS, APRI, and FIB-4 and lower platelet-to-spleen ratio as compared with patients with low risk EV (Table 4). Multivariate analysis disclosed that LSPS only (OR 1.456, 95 % CI 1.083–1.957; *P* = 0.013) was independently associated with a high risk of EV in CLD.

**Table 4:** Characteristics of patients with and without high risk EV

Characteristic	Low risk (n = 29)	High risk (n = 10)	*P* value
Age (years)	65 (13–90)	54 (14–74)	0.145
Male	17 (59)	8 (80)	0.279
Body mass index	24.3 (17.3–41.1)	25.0 (18.5–30.2)	0.530
Etiology			
NAFLD/NASH	4 (14)	3 (30)	0.500
HBV	10 (34)	0 (0)	0.083
PBC	4 (14)	1 (10)	0.811
ALD	6 (21)	4 (40)	0.432
Other	5 (17)	2 (20)	0.778
Platelet count (×10^9^/l)	118 (39–683)	55 (34–99)	0.039
AST (IU/l)	30 (17–146)	42 (15–120)	0.326
ALT (IU/l)	24 (8–86)	29 (9–123)	0.367
Albumin (g/dl)	3.9 (2.7–4.6)	3.5 (3.0–4.2)	0.055
T.Bil (mg/dl)	1.0 (0.5–7.5)	2.4 (0.4–7.1)	0.014
PT%	78 (40–95)	64 (27–92)	0.004
LS (kPa)	7.7 (3.5–75.0)	24.9 (9.1–75.0)	0.004
Spleen size (cm)	10.3 (6.1–24.0)	13.9 (7.7–16.6)	0.050
LSPS	1.0 (0.1–9.5)	5.5 (1.2–20.0)	<0.001
APRI	1.1 (0.1–4.8)	2.4 (0.8–4.0)	0.026
FIB-4	4.2 (0.1–12.0)	8.4 (1.2–10.9)	0.044
Platelet-to-spleen ratio	1092 (225–5591)	407 (220–770)	0.003

Values are expressed as median (range, minimum–maximum) or n (%)

The performance of noninvasive parameters for identifying high risk EV, including AUC, optimal cutoff value, sensitivity, specificity, PPV, NPV, and accuracy, is summarized in Table 5. Calculated AUCs were 0.859 for LSPS, 0.833 for platelet count, 0.817 for platelet-to-spleen ratio, 0.807 for LS, 0.762 for APRI, and 0.716 for FIB-4. Although LSPS had the best discrimination for high risk EV, there were no significant differences between the AUC of LSPS and those of platelet count, platelet-to-spleen ratio, LS, or APRI. The optimal LSPS cutoff value of 2.2 provided a sensitivity of 90.0 %, specificity of 72.4 %, PPV of 52.9 %, NPV of 92.5 %, and accuracy of 76.9 % (Table 5).

**Table 5:** Performance of noninvasive parameters for identifying high risk EV among 39 patients complicated with EV

Characteristic	Cutoff	AUC (95 % CI)	Sensitivity (%)	Specificity (%)	PPV (%)	NPV (%)	Positive Likelihood ratio	Negative Likelihood ratio	Accuracy (%)
LSPS	4.5	0.859 (0.736–0.981)	70.0	86.2	63.6	89.3	5.08	0.35	82.1
Platelet count (×10^9^/l)	100	0.833 (0.707–0.958)	100.0	65.5	50.0	100.0	2.61	0.00	74.3
Platelet-to-spleen ratio	990	0.817 (0.687–0.947)	100.0	65.5	50.0	100.0	2.90	0.00	74.3
LS (kPa)	12.0	0.807 (0.674–0.940)	80.0	75.9	53.3	91.7	3.31	0.31	76.9
APRI	1.7	0.762 (0.609–0.915)	80.0	72.4	50.0	91.3	2.90	0.28	74.4
FIB-4	5.0	0.716 (0.529–0.902)	90.0	58.6	42.9	94.4	2.18	0.17	66.7

## Discussion

Although LSPS has shown promise as a predictive marker of EV and/or high risk EV (Berzigotti et al. 2013; Kim et al. 2010; Shibata et al. 2015), additional trials are needed to validate its clinical utility. The present study confirmed the diagnostic accuracy of LSPS for detecting EV and high risk EV in patients with CLD of various etiologies. Moreover, LSPS was well correlated with EV grade (*P* < 0.001). The diagnostic accuracy and AUC of LSPS for identifying EV were 84.3 % and 0.821, respectively, and multivariate analysis revealed that LSPS had the highest performance in identifying EV in CLD. Hence, the current study on patients with CLD of various etiologies validated the usefulness of LSPS found in our prior report on chronic HCV infection (Shibata et al. 2015). The EV cutoff value was low in this study, indicating that those patients who displayed a lower value were presumed not to be complicated with EV based on the NPV. However, clinicians should recommend further endoscopic evaluation for CLD patients scoring greater than the LSPS cutoff value to evaluate for EV, even though endoscopy is an unpleasant procedure that carries rare, but serious, complications.

Simple, reliable, noninvasive methods are sought to better identify high-risk cases of F2/F3 EV or F1 EV with red color signs prior to endoscopy and thus avoid variceal bleeding. The diagnostic accuracy of LSPS as determined by the AUC in the current study was similar to that of earlier published data (Kim et al. 2010; Shibata et al. 2015).

This investigation had several limitations. It was retrospective in design. Longer follow-up will be required to evaluate the prediction of EV and bleeding EV, especially in patients without high-risk EV in CLD, to overcome a possible selection bias in this study. The relatively small sample size may have influenced the results as well; for example, the LS of alcoholic LD was higher than those of other etiologies (*P* < 0.001), although the number of alcoholic LD patients was too small for any definite conclusions. A further study will be needed to clarify which values of LS (including that for LSPS) can predict EV according to etiology in CLD. Moreover, it was difficult to determine how many patients could be spared endoscopy, could avoid unnecessary endoscopy, or had received unnecessary endoscopy with this study model since approximately 40 % of subjects were excluded due to reasons such as an IQR of greater than 30 %. During the study period, 4462 patients received endoscopy at our institution, 251 of whom (5.6 %) had evidence of varices by endoscopy. Fifty-seven of 251 patients with EV (22.7 %) exhibited high risk EV. As patients with obesity or ascites are not good for candidates for TE, other surrogate fibrosis biomarkers, such as Mac-2-binding protein (Kuno et al. 2013; Umemura et al. 2015), are being considered for these individuals.

## Conclusion

This investigation validates LSPS as a potent, noninvasive method for predicting EV and high risk EV in Japanese patients with CLD. Clinicians should recommend those patients with CLD who show higher values of LSPS to undergo further endoscopic examination.

## Additional file

10.1186/s40064-016-2708-1 Frequency of LS IQR < 30 % vs. that of LS IQR ≥ 30 % for each etiology. Frequencies of LS IQR < 30 % (dark gray) and those of LS IQR ≥ 30 % (light gray) according to etiology in all subjects (n = 835). The frequency of LS IQR < 30 % for HCV is significantly lower than those of other etiologies. There are no differences among the other etiologies of CLD.

## Abbreviations

APRI: aspartate aminotransferase-to-platelet ratio index
AUC: area under the ROC curve
CIs: confidence intervals
CLD: chronic liver disease
EV: esophageal varices
kPa: kilopascals
LS: liver stiffness
LSPS: LS-spleen diameter-to-platelet ratio risk score
NAFLD: non-alcoholic fatty liver disease
NASH: non-alcoholic steatohepatitis
NPV: negative predictive value
PPV: positive predictive value
ROC: receiver operating characteristic
TE: transient elastography
US: ultrasonography
