# Supplementary figures and images for: Liver stiffness-spleen size-to-platelet ratio risk score detects esophageal varices in chronic liver disease
Source: Springerplus. 2016 Jul 7;5(1):998. doi: 10.1186/s40064-016-2708-1 (PMC4936997; doi:10.1186/s40064-016-2708-1)

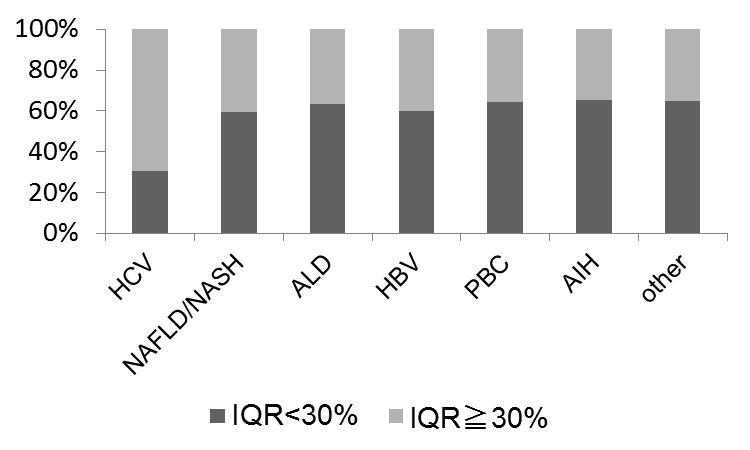

Supplement: Supplementary file 1 — 10.1186/s40064-016-2708-1 Frequency of LS IQR < 30 % vs. that of LS IQR ≥ 30 % for each etiology. Frequencies of LS IQR < 30 % (dark gray) and those of LS IQR ≥ 30 % (light gray) according to etiology in all subjects (n = 835). The frequency of LS IQR < 30 % for HCV is significantly lower than those of other etiologies. There are no differences among the other etiologies of CLD. [file 40064_2016_2708_MOESM1_ESM.tif]
